# Shutting the studio: the impact of the Covid-19 pandemic on architectural education in the United Kingdom

**DOI:** 10.1007/s10798-022-09765-y

**Published:** 2022-08-29

**Authors:** Robert Grover, Alexander Wright

**Affiliations:** Department of Architecture and Civil Engineering, University of Bath, BA2 7AY Bath, UK

**Keywords:** Architectural education, Design studio, Remote learning Covid-19

## Abstract

The Covid-19 pandemic instigated a rapid shift to remote learning in schools of architecture in the United Kingdom. Through the largest survey of its kind of architectural students and tutors in the UK, this research compares experiences in the physical design studio and its remote equivalent. The context of the pandemic provided a unique opportunity to survey a range of cohorts, at different stages in their architectural education to compare these two modes of studio delivery. The findings show a fall in student satisfaction after the move to remote learning in every metric assessed. Peer interaction and support were particularly effected. More formal teaching interactions, such as reviews, crits and tutorials, also suffered but to a lesser extent. For teaching staff, some small improvements in the working environment were observed as well as organisational factors. However, these small gains were outweighed by the negative changes. The research suggests that despite the replication of teaching activities digitally, the situated learning of design education and the facilitation of informal learning scenarios are critical components of design education. This research contributes to the ongoing characterisation of architectural education’s signature pedagogy and suggests that for effective remote learning, design studio education must be reconceptualised, and alternative pedagogies embraced. This can direct educators looking to develop remote design studio learning as well as highlighting areas in which the traditional model of architectural education may be enhanced.

## Introduction

This research sets out to compare staff and students experiences of in-person and remote learning in architectural education. The enforced closure of physical design studios during the Covid-19 pandemic provided a unique cohort of students who directly experienced both in person and remote forms of delivery, within a single academic year, and in every architecture programme in the UK. This research describes the findings of a nationwide survey.

The design studio and its associated pedagogy has been widely adopted in the UK as the primary means of educating architects (Vowles et al., 2012). Its physical context has been recognised as an integral component to the profession’s “signature pedagogy” in which future architects embed cognitive and performative professional skills (Shulman, 2005). The validation provided by the Royal Institute of British Architects (RIBA) explicitly requires that all validated RIBA part 1 and part 2 courses should have design projects constitute a minimum of half of all assessment (RIBA, 2014). Despite the increasing prevalence of virtual design processes, the spatial dimension of the studio has remained central to the pedagogy of the architectural profession (Corazzo, 2019; Brown, 2020). The shutting of design studios and university buildings in the UK, offered a chance for this cohort of students to reflect on the value of the physical studio and highlight its shortcomings and benefits.

## Literature Review

Studies into the design studio have frequently referred to Donald Schön’s work on the design studio (Schön, 1985) which focussed on the formal interactions between student and tutor in this setting. His observations and case studies framed an understanding of the cognitive processes taking place during design. Subsequent scholars have sought to challenge his conception of the design studio, highlighting a range of complex behaviours, experiences and interactions which frame learning in this context (inter alia Stevens 2002; Webster, 2004; Vowles, 2005; Quinlan et al., 2007; Webster, 2008; Corazzo, 2019; Brown, 2020). The studio has also been identified as a site for power asymmetries and exclusivity (inter alia Datta 2007; Webster, 2007) or a space in which norms are habitually reproduced (inter alia Dutton 1987; Banham, 1991; Stevens, 2002). A range of studies have attempted to replicate the environment of the design studio remotely, however this remains at the level of individual case studies (inter alia Abbasi et al., 2018; Lahti & Seitamaa-Hakkarainen 2014; Salman et al., 2017; Lotz et al., 2019; Jones et al., 2020).

Donald Schön’s work on the Design Studio (Schön, 1985) focussed on the formal interactions between student and tutor in the studio setting. Teaching took place through the tutor demonstrating their own internal reflective actions to the student by simulating their design process through drawing. Schön emphasised the performative nature of design studio teaching in which the knowledge of tutors is made specific to the student’s situation through such demonstration and simulation (Goldschmidt et al., 2010).

Scholars have critiqued Schön’s limited conception of learning and emphasised the complex, interdependent pedagogy of the studio (Webster, 2008; Brown, 2020). While the pedagogic benefits of Schön’s approach are questionable, particularly the assumed automatic learning through observation and the downplaying of individual student lived experiences (Webster, 2004), his work highlights the fundamental inheritance of in-situ learning in architecture. For example, the various constructions of studio-supervisor offered by Belluigi (2016) assume a one-to-one relationship facilitated by the physical design studio. In a study by Quinlan et al., (2007), they suggest that personal qualities of the tutor (particularly attentiveness and ‘presence’) are highly desirable by students. Demonstrating both credibility and authenticity are required by tutors to build meaningful relationships with students over time, emphasising the experiential qualities afforded by an in person, place-based, education.

In a meta-study of design studios in architecture and the arts, Corazzo (2019) identified six key themes that the material space of the design studio enabled: a place to make artefacts; a bridge between academic and professional contexts; to provide meaning to educational activities; to enable or constrain experience and interaction; to provide the background to learning; and to shape disciplinary identities. An implicit belief in a resultant “studio culture” has governed the pedagogic approaches of architectural education institutions in the UK despite a limited definition of its parameters (Vowles et al., 2012). Nevertheless, the design studio is widely accepted to be a rich learning medium which nurtures peer interaction and independent learning (McClean, 2009).

Scholars have frequently cited the importance of social structures within the design to enhance learning. Vowles (2005) identifies the importance of social interaction, communicating with peers whether verbally or visually, to both the processes of design and education. Moreover, the social processes of the studio, the formation of the community and the norms that are validated through shared practice contribute to a “thickly authentic” environment (Shaffer & Resnick, 1999). In the studio this translates to a coherence between activities which reproduce the norms of the wider profession (Shaffer, 2003). The intense culture of the design studio also facilitates strong social bonds. McClean (2009) notes the capacity of the design to enable pastoral support from both peers and tutors. Students described a “peer dynamic”, and compared the studio to a “home”, a place which offered peer support and pastoral care (McClean, 2009, p.207, p.207).

The relationship between space and learning is fundamental to the character of the design studio. However, this connection has been severed in much of the literature on learning in higher education (Neary et al., 2009). As a place of both formal and informal learning activities, it bridges the divide between these two settings (Boys, 2010, p.2, p.2). The design studio is not only the backdrop for learning and social interaction but is implicit in these activities. Drawing from the work of Lefebvre & Nicholson-Smith (1991), McGregor (2004) asserts that the place of learning might be understood through the “materially embedded practices” and space itself is essential to the production and reproduction of social structures (Corazzo, 2019). As numerous scholars have suggested, this has the potential for unequal power dynamics that perpetuate existing hierarchies (Datta, 2007; Webster, 2007), as well as uncritically reproducing behaviours of the profession (Dutton, 1987; Banham, 1991; Stevens, 1995).

Attempts to replicate the design studio experience remotely have often focussed on generating digital spaces where students can share work and interact asynchronously (Lotz et al., 2015). While there have been reported success in virtual online studios (Abbasi et al., 2018; Lahti & Seitamaa-Hakkarainen 2014; Salman et al., 2017; Lotz et al., 2019; Jones et al., 2020) and blended approaches (Mohammed, 2017; Rodriguez et al., 2018), these typically utilise formal online spaces (such as e-portfolios or online databases) to simulate peer interaction.

In UK architectural education, the research conducted by Hannah Vowles (Vowles et al., 2012), perhaps offers us the most recent insight into design studio culture. The report highlights the perceived value of developing a “studio culture” in the UK. Despite the advancement of digital technologies, the physical studio remained critical as a place of peer learning and student staff interaction.

In the context of the international pandemic, it is also helpful to differentiate between genuine online learning and the emergency remote delivery that was observed during this period recognised by Hodges (2020). As the authors assert, designed online learning create learning ecosystems to support students through building communities and support networks. By contrast, emergency remote teaching tends to aim to make instructional resources reliably accessible. While the authors are unaware of any studies which take a sample of students familiar with the physical design studio and compare this with a move to remote learning, it must be understood in the context of the unplanned and instantaneous shift to online delivery. Nevertheless, this provides a unique opportunity to enhance both remote and face-to-face learning through a comparative understanding of the successes of each mode of delivery.

## Aims of the research

This research aimed to compare student and staff experiences of in person design studio teaching and remote delivery. It sought to establish satisfaction with each mode of delivery to highlight areas to be addressed by educators. Through this comparison, it also intended to identify the key characteristics that are valued by students and staff in design led architectural education.

## Methodology

The Covid-19 pandemic, which forced the closure of schools of architecture in the UK, provided a unique opportunity to compare the experiences of a single cohort of students in two contrasting learning environments (in-person and remote). The scale and totality of the closures provided a large and unique population of students who had experience of both modes of delivery sequentially.

The research used an internet survey methodology to capture satisfaction with their architectural education, before and after the closure of the studios. The research sought to gather responses from a population of staff and students in all RIBA validated architecture schools in the UK. An internet survey had the advantage of being fast, easy to distribute, convenient and easy to complete. It also allowed for increased anonymity and accuracy through the reduction of human error (Cohen et al., 2017). It allowed customisation of the release of the survey for different schools of architecture; in all cases the survey was released to students in the days following their final submissions. The survey was hosted on the online platform *Jisc Online Surveys* (Jisc, 2022).

### Sampling

The sample was an “unrestricted sample” (Watt, 1997) from the target population of all students and staff from RIBA validated schools of architecture in the UK. 798 students and 120 tutors from 29 UK universities responded to the survey. The survey was distributed through members of the Standing Conference of Heads of Schools of Architecture (SCHOSA) to administer in their respective schools. The surveys were distributed immediately after students’ final submissions, with each school representative responsible for this timing. All students were either taking their first degree (RIBA Part 1) or RIBA Part 2 (typically an undergraduate masters degree).

### Survey questionnaire

The online survey was via a questionnaire which utilised two primary different question types. These are outlined in Table 1.

**Table 1:** Question types and uses

Question type	Use
Multiple choice	Used for questions with limited number of possible options (such as types of teaching activity) or questions with predefined categorisation (such as gender or ethnic group).
Semantic differential items	Used for comparative questions on relevant attributes. Individual items focussed on specific activities or event.
Limited text	Used for questions requiring continuous scales (such as time or distance) or short answer responses (such as which University the respondent attended).

The primary mode of data collection was through labelled semantic differential items. This labelling has been shown to yield most accurate response among a general population to avoid confusion between unlabelled responses (Garland, 1990).

Each theme was broken into a series of unambiguous items Each item consisted of an object relating to an overall theme and a ‘response scale’ (Johns, 2010), in most cases asking for the respondent’s level of satisfaction with each metric. A five-point scale with a neutral mid-point was used in both surveys for all semantic differential questions as these are more typically understood by participants than more granulated scales (Garland, 1990). Each object was intensity free (i.e. did not include a positive or negative assertion or a magnitude). They were considered to be “concrete”, a singular unambiguous concept. Each object was assessed through a single, relevant, and concrete attribute requiring only a single item (Rossiter, 2002).

While satisfaction was the primary attribute assessed, data are presented as positive vs. negative responses in the findings. The themes, items and attributes to which staff and students responded are presented in Tables 2 and 3. A “non-applicable” option was also provided in every case.

**Table 2:** Themes, items and attributes on semantic differential responses on student questionnaire

Themes and objects	Positive attributes	Neutral attribute	Negative attributes
**Environment**: ­ **in the architecture studio** ­ **remotely**			
­ Workstation ergonomics (desk, chair, comfort etc.) ­ Lighting, heating and ventilation ­ Acoustics and noise control ­ Layout space (for Drawings and models) ­ Storage space ­ Plotting, printing and scanning facilities ­ Network connectivity ­ Access to software ­ Access to hardware ­ IT services	Very satisfied / Fairly satisfied	Neither satisfied nor dissatisfied	Fairly dissatisfied/ Very dissatisfied
**Types of learning activity**: ­ **in the architecture studio** ­ **remotely**			
­ Individual tutorials when you see your tutor on your own ­ Group tutorials when you see your tutor together with one or more other students ­ Lectures ­ Accessing on-line resources ­ All types of architecture reviews/crits/juries	Very satisfied / Fairly satisfied	Neither satisfied nor dissatisfied	Fairly dissatisfied/ Very dissatisfied
**Types of feedback**: ­ **in the architecture studio** ­ **remotely**			
­ Feedback from individual tutorials ­ Feedback from group tutorials ­ Feedback from all types of reviews/crits/juries	Very satisfied / Fairly satisfied	Neither satisfied nor dissatisfied	Fairly dissatisfied/ Very dissatisfied
**Support**: ­ **in the architecture studio** ­ **remotely**			
­ Non-project related support and advice from tutors (pastoral advice) ­ IT and technical support services ­ Access to central student support services (counselling, finance, immigration) ­ Access to the Students’ Union support services	Very satisfied / Fairly satisfied	Neither satisfied nor dissatisfied	Fairly dissatisfied/ Very dissatisfied
**Learning from others**: ­ **in the architecture studio** ­ **remotely**			
­ Seeing the work of other students in your year ­ Seeing the work of students in other years ­ Advice and feedback from other students in your year ­ Technical help from other students in your year (CAD, IT, model making help etc.) ­ Sharing resources (models, CAD info, equipment, books etc.) ­ Observing other students’ reviews and tutorials ­ Working in a team	Very satisfied / Fairly satisfied	Neither satisfied nor dissatisfied	Fairly dissatisfied/ Very dissatisfied
**Overall satisfaction**: ­ **in the architecture studio** ­ **remotely**			
­ Overall satisfaction	Very satisfied / Fairly satisfied	Neither satisfied nor dissatisfied	Fairly dissatisfied/ Very dissatisfied

**Table 3:** Themes, items and attributes on semantic differential responses on staff questionnaire

Themes and items	Positive attributes	Neutral attribute	Negative attributes
**Environment**: ­ **in the architecture studio** ­ **remotely**			
­ Workstation ergonomics (desk, chair, comfort etc.) ­ Lighting, heating and ventilation ­ Acoustics and noise control ­ Layout and working space ­ Storage space ­ Printing, plotting and scanning facilities ­ IT services ­ Access to software ­ Access to hardware ­ Network connectivity	Very satisfied / Fairly satisfied	Neither satisfied nor dissatisfied	Fairly dissatisfied/ Very dissatisfied
**Student engagement**: ­ **in the architecture studio** ­ **remotely**			
­ Students’ preparation for tutorials ­ Students’ preparation for reviews/crits/juries ­ Students’ engagement in individual tutorials ­ Students’ engagement in group tutorials ­ Students’ engagement with reviews/crits/ juries ­ Student progress between tutorials ­ Students’ punctuality ­ Students’ preparation of the final submission ­ Quality of student drawings and 2D media ­ Quality of student models and 3D media	Very good / Fairly good	Neither good nor poor	Fairly poor/ Very poor
**Teaching facilitation**: ­ **in the architecture studio** ­ **remotely**			
­ Providing design feedback on the students’ proposals in tutorials ­ Providing design feedback on the students’ proposals in reviews/crits/juries ­ Exploring design options with the student in tutorials ­ Discussing or exploring precedents in tutorials ­ Assessing the students’ state of well-being ­ Providing pastoral (advice on ways of working, time management, careers, etc.) ­ Keeping to the scheduled time for each tutorial	Very easy / Fairly easy	Neither easy nor difficult	Fairly difficult/ Very difficult
**Feedback and assessment procedures**: ­ **in the architecture studio** ­ **remotely**			
­ Verbal feedback from formal reviews/crits/juries with external critics ­ Written/drawn feedback from formal reviews/crits/juries with external critics ­ Verbal feedback from design tutorials ­ Written/drawn feedback from design tutorials ­ Marking student design reports/projects/portfolios ­ Moderation of student design reports/projects/portfolios	Very satisfied / Fairly satisfied	Neither satisfied nor dissatisfied	Fairly dissatisfied/ Very dissatisfied
**Opportunities for peer to peer student learning**: ­ **in the architecture studio** ­ **remotely**			
­ Seeing the work of other students in their year ­ Seeing the work of students in other years ­ Giving and receiving advice and feedback from other students in their year ­ Receiving technical help from other students in their year (CAD, IT, model making help etc.) ­ Sharing resources (models, CAD info, equipment, books etc.) ­ Observing other students’ reviews and tutorials ­ Students working in teams	Very good / Fairly good	Neither good nor poor	Fairly poor/ Very poor
**Overall satisfaction**: ­ **in the architecture studio** ­ **remotely**			
­ Overall satisfaction	Very satisfied / Fairly satisfied	Neither satisfied nor dissatisfied	Fairly dissatisfied/ Very dissatisfied

There were also a range of free text responses which asked for advantages, disadvantage, challenges and opportunities for remote working as well as any further comments. These were included on both the tutor and student questionnaires.

### Analysis

The findings analyse the cumulative responses to individual items and treats the data as ordinal (Boone & Boone, 2012). Data are presented in mostly graphical form. The semantic differential items were assumed to be non-parametric and are presented using descriptive statistical measures such as cumulative responses, relative difference between related items (i.e. the difference as a proportional increase or decrease of the original metric) and total satisfaction. When using terms such as satisfaction, the survey sums all positive responses (“fairly satisfied” and “very satisfied”) without weighting them which would imply magnitude or interval data.

Over 4000 individual qualitative free text responses were analysed. Thematic analysis was used adopting the six stages described by Braun and Clarke (2006). Familiarisation was achieved through reading through the comments, and these were then categorised into initial codes. In many cases, a single response might contain multiple pieces of coded data. Codes were then collated into themes. These themes were then reviewed once all data had been coded. This enhanced the definition of each theme. Finally, the report sought to concisely capture these themes. Coding was done at a semantic level (i.e. hidden meaning in the was assumed). This was appropriate to the concise and precise nature of the comments. The themes were quantified (Boyatzis, 1998) to identify the most common trends.

### Research ethics

All data were collected anonymously, and responses contained no data which could identify individuals. Individuals were made aware of the data storage practices at the start of the research and it was mad clear they could withdraw from the survey at any point before submitting. Due to the anonymous collection procedure, data could not be removed relating to an individual after submission. Individuals could not be directly nor indirectly identified so the data were excluded from the UK General Data and Protection Regulation (UK GDPR).

### Limitations and bias

Scholars have noted methodological issues related to sampling in web-based surveys, not least the tendency for self-selection and accessibility (Duffy, 2002). This is largely negated by the population profile, all of whom have access to the internet and computers through their university membership and are frequently surveyed from within and without their institutions.

The semantic differential approach has significant advantages over Likert type responses. By rating an object through a given attribute, rather than being asked to agree or disagree with a statement, the problems of acquiescence bias are negated (Friborg et al., 2006). Despite introducing greater cognitive load on participants, this has still been shown to produce more reliable responses (Friborg et al., 2006). Bipolarity of terms is necessary to a semantic differential test and accordingly, clear, relevant terms with clear oppositions were chosen (satisfaction / dissatisfaction, good / poor, easy / difficult) (Verhagen et al., 2015).

Items were asked in a consistent order (first the physical design studio was rated, then the remote design studio). While studies have noted the contextual influence of items (the preceding item can influence the response to the latter) (Landon Jr,, 1971), in this instance, due to the repetition of questions, consistent ordering would reduce the cognitive load on participants and reduce errors (Friborg et al., 2006). The work is presented as a change in attributes across the design studio and the remote studio, with the design studio being asked first acting as a consistent point of comparison for respondents for responses (Landon Jr,, 1971).

The timing of the survey also limits how the results can be interpreted. It was circulated at the end of term, a point at which students had immediately finished their studies. Without some reflective distance, the responses may have used as a proxy for widespread anger and disruption caused by the pandemic, as well as dissatisfaction with assessments. Moreover, the results contrast the students’ current experience of remote learning with their remembered experience of live teaching.

## Findings

### Teaching in the physical design studio is considered integral to architectural education by students and staff

**Fig. 1:**
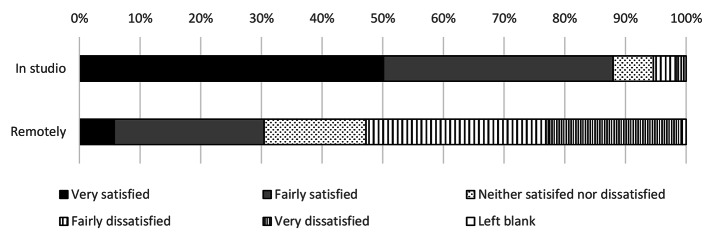
Overall student satisfaction with the architectural studio and working remotely

**Fig. 2:**
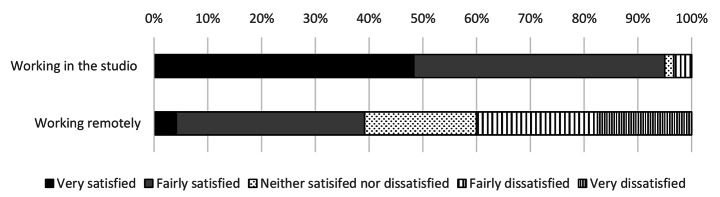
Tutors’ satisfaction with modes of delivery of architectural education

Before the move to online learning, 88% of all students were either fairly satisfied or very satisfied with their experience of the design studio (Fig. 1). This fell to 30% satisfaction after the studios were closed, a relative fall of 65%. Total satisfaction fell in the move to online learning across every metric surveyed. Only 7% of students preferred remote delivery to face to face learning. This also was true of staff who showed a marked drop in satisfaction comparing the two modes of delivery (Figs. 2) and 4% of staff preferred remote delivery to face-to-face teaching. This was despite only 25% of students surveyed being offered a permanent workspace in the studio pre-pandemic.

**Table 4:** Representative quotes of the integral nature of the design studio to design education

Student comments	Staff comments
“Online school is not the same. There is not the same connection between tutor and student. Harder to communicate ideas. IT IS NOT THE SAME.” (1st year, Part 1 student) “If working remotely for the next year is an option, I would definitely not chose it. Working at home has created new challenges such as boredom, dissatisfaction which drastically decreases motivation to work.” (2nd year, Part 1 student) “If there is remote learning next year I will be forced to take a year out. I don’t have the resources to do the work to my best ability and I’m not getting enough support from the university” (2nd year, Part 1 student) “Doing architecture cannot be done virtually, to practice architecture is to physically connect, create, discuss which cannot be imitated virtually. Nothing can replace the atmosphere of a review/ studio/ show where chance encounters between years and staff can take place.” (3rd year, Part 1 student) “I find it really concerning that remote working for architecture degrees may continue for longer than necessary. It works for lectures but definitely not for studio reviews and tutorials. The lack of contact with other students and tutors is a major disadvantage in my opinion. We students simply don’t have the facilities or space at home to complete and architecture project.…Also working remotely with minimal contact with other students is a massive cause for concern particularly when regarding students’ mental health (on top of the already existing issue of degrees of architecture’s negative affect on mental health).” (1st year, Part 2 student) “To stress, the studio as a physical space and the amenities that it comes with are CRUCIAL to my architectural education. Present standards of remote working have had a serious negative impact on my current and future architectural education…Communication and collaboration are critical conditions in Architecture, how is this supposed to be achieved in isolation?” (1st year, Part 2 student) “I think studio culture is a massive thing from architecture students which isn’t the same as people studying courses that are just lectures who may find remote working easier.” (2nd year, Part 1 student)	“The nature of the architecture studio as a critical learning environment in physical space is fundamental to the future of the discipline. While we are all coping in a sense to support our students in a very challenging time… it is only a precarious holding position. There has never been a more fundamental registering of the true value and importance of the studio environment and culture than now…when it is inaccessible.” (Undergraduate and MArch tutor) “This is a fundamental issue. It lies at the very heart of the discipline. Studio culture is what a university can offer. Other agencies can offer remote/online teaching. Only a university can offer the studio, the library, the refectory, the lecture theatre. Lose these and you lose students.” (Undergraduate and MArch tutor) “Esprit de corps of studio working lost. Students are isolated and do not enjoy or benefit from a studio culture. No contact with other tutors and tenuous contact with critics in revues. The three-way dialogue of a review is lost. Difficulty communicating drawn comments on student work online through lack of hardware and limitations of TEAMs software. A sense of isolation from the collegiate body.” (Undergraduate tutor)

### Peer learning and support networks were particularly affected by the closure of design studios

**Fig. 3:**
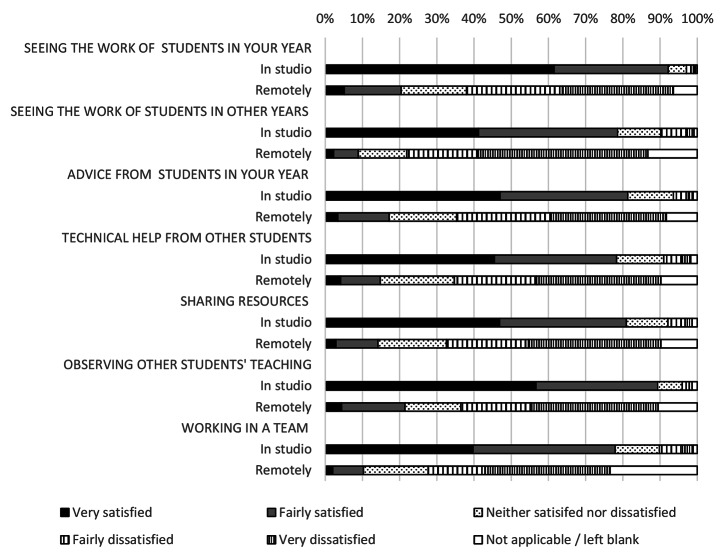
Student satisfaction with peer learning in the architecture studio and working remotely

**Fig. 4:**
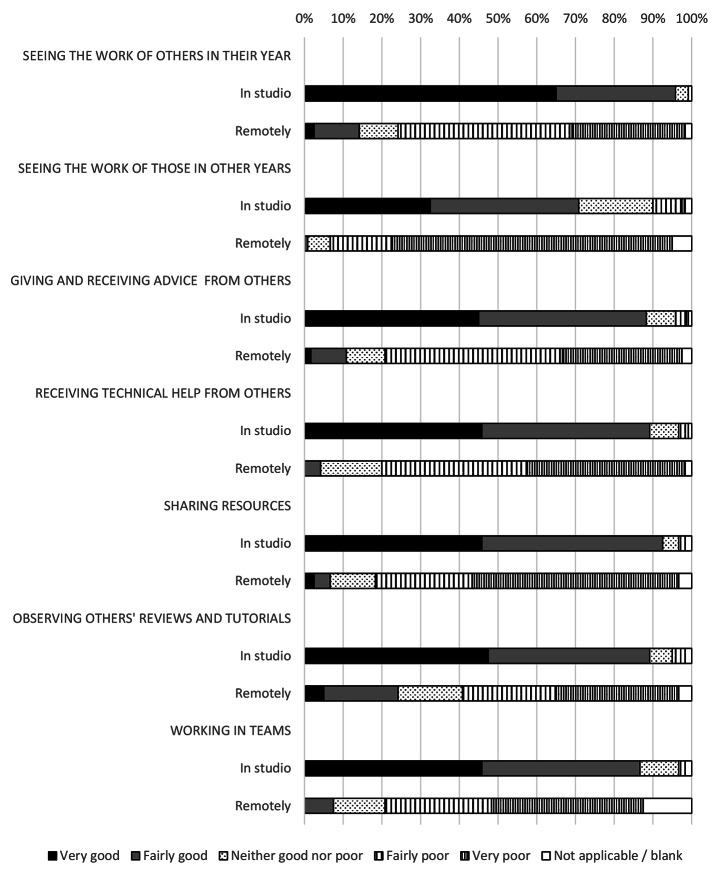
Tutors’ perceived quality of peer learning mechanisms in the architecture studio and working remotely

The move to remote learning severed peer networks and support systems (Fig. 3). Students described a lack of community, poor motivation and being unable to benchmark their progress. For example, total satisfaction with seeing the work of students in their year group fell by 72% following the move to online learning; the largest fall in any metric. These concerns were echoed by teaching staff (Fig. 4). The loss of peer support networks also had impacts on student mental health and wellbeing; the move to remote studio teaching inducing a sense of isolation in many of the respondents.

In qualitative comments, 51% of students cited the lack of peer learning and support as a major disadvantage. 28% of students said their ability to learn from their peers had been negatively impacted. Often this was through the loss of being able to see the work of other students. A similar number cited the loss of peer support. Many talked about the loss of *studio culture*, lack of informal learning experiences and the motivational support provided by other students. The loss of a sense of community was also a major concern.

39% of staff described how the remove to remote tutoring had impacted negatively various aspect of “studio culture”. 8% of staff used this term directly while other spoke about the loss of peer interaction (16%) lack of informal engagement (8%) and low levels of social interaction (5%). The loss of the professionalism of the studio, the notion of a “shared experience” and the ability for students to see the work of their peers were also mentioned.

**Table 5:** Representative quotes about peer learning

Student comments	Staff comments
“Not having friends/other students surrounding you. We’re taught from the very early stages of our architecture education that working within the studio is highly important to benefiting our studies. “Studio culture” is often referenced when talking about architecture education. It’s a hard enough course to be doing, and without the moral support, help and advice of your peers, it’s been very difficult to ‘continue as normal’ which I feel is being expected of us.” (2nd year, Part 2 student) “I miss studio culture, being able to bump into fellow students and tutors. See random models as you walk around uni. Being inspired by other people’s work and helping each other. Also being able to gauge other people’s progress to see if you are on track.” (2nd year, Part 1 student) “Working remotely as a whole is a disadvantage, it dulls and plateaus creativity, it doesn’t allow the spontaneous conversations about projects which could better your work that the studio provides.” (1st year, Part 2 student) “I don’t want to work remotely from home, honestly it’s the worst experience anyone can have. It’s too distracting, there’s no motivation and I can’t see other people’s work. How is this a good solution? How am I supposed to learn like this? … I fear this will be the main reason I will drop out of Architecture, because no one wants to work like this.” (1st year, Part 1 student) “Any group work next year will be very challenging both to set up and get to know the group and to organise effectively without meeting face to face.” (1st year, Part 2 student)	“[The biggest disadvantage was] the lack of studio culture- social engagement, learning from each other, taking pride and ownership of space and work.” (MArch tutor) “[The biggest challenge was] students not supporting each other as simply as they could in a studio. I found a way for students to upload their work so others could see it, I think this was important to inform the other students where they should be in terms of progress. For me first year is an extremely important year for social networking and adopting a studio environment and a productive one. I doubt that can be recreated.” (Undergraduate tutor) “Studio experience is an identifiable loss. With everything that goes along with it: interpersonal relationships, friendships, camaraderie, as well as peer to peer learning, spatial engagement, etc.” (Undergraduate and MArch tutor)

### There was no significant difference in the demographic groups surveyed

The survey found that the demographic groups of gender (Fig. 6), ethnic group (Fig. 7), study status (home, EU or overseas) (Fig. 8), were not statistically significant different in changes in overall satisfaction.

**Fig. 5:**
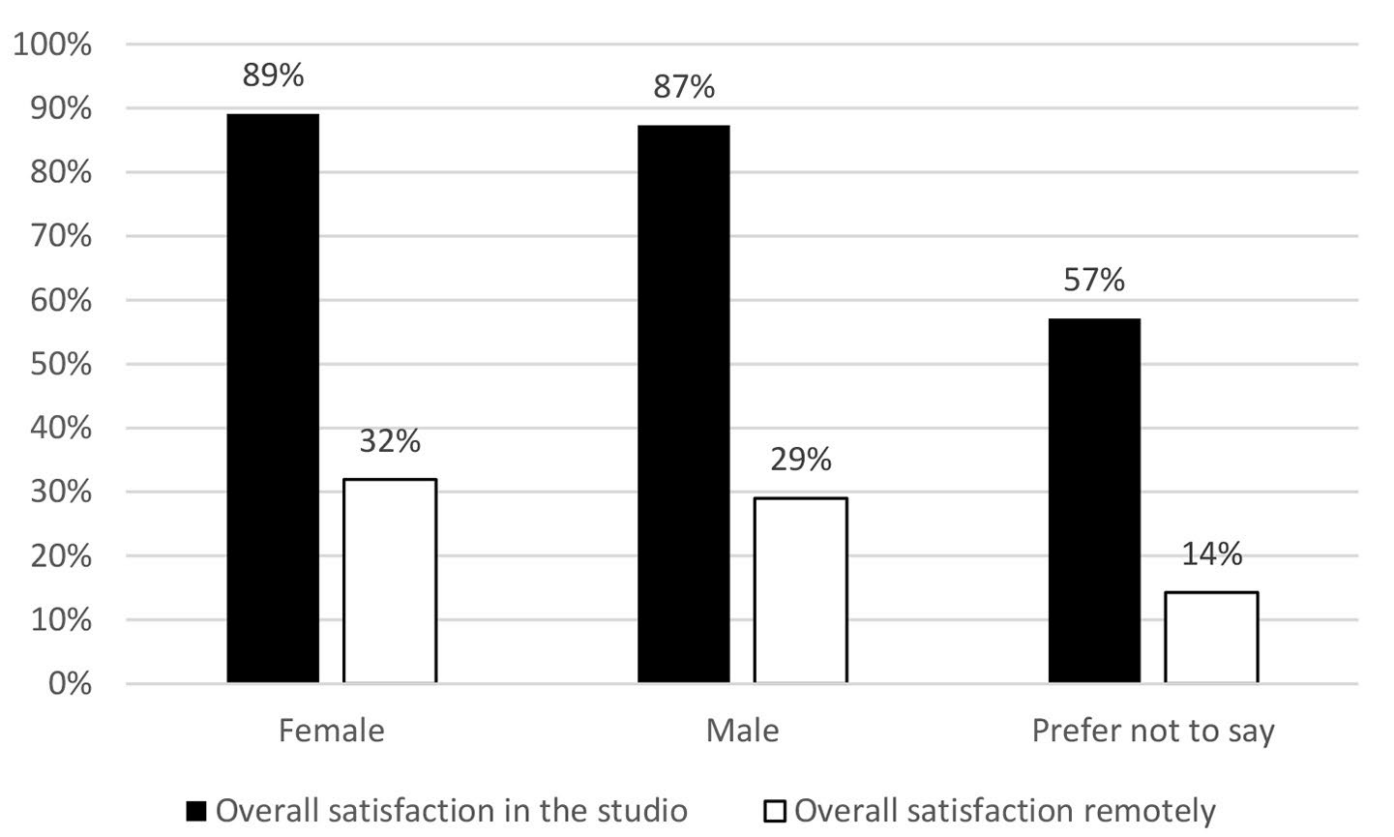
Change in overall satisfaction in different genders

**Fig. 6:**
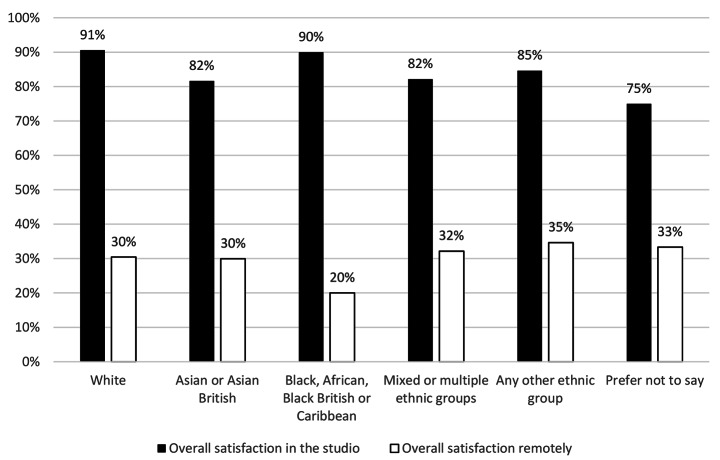
Change in overall satisfaction in different ethnic groups

**Fig. 7:**
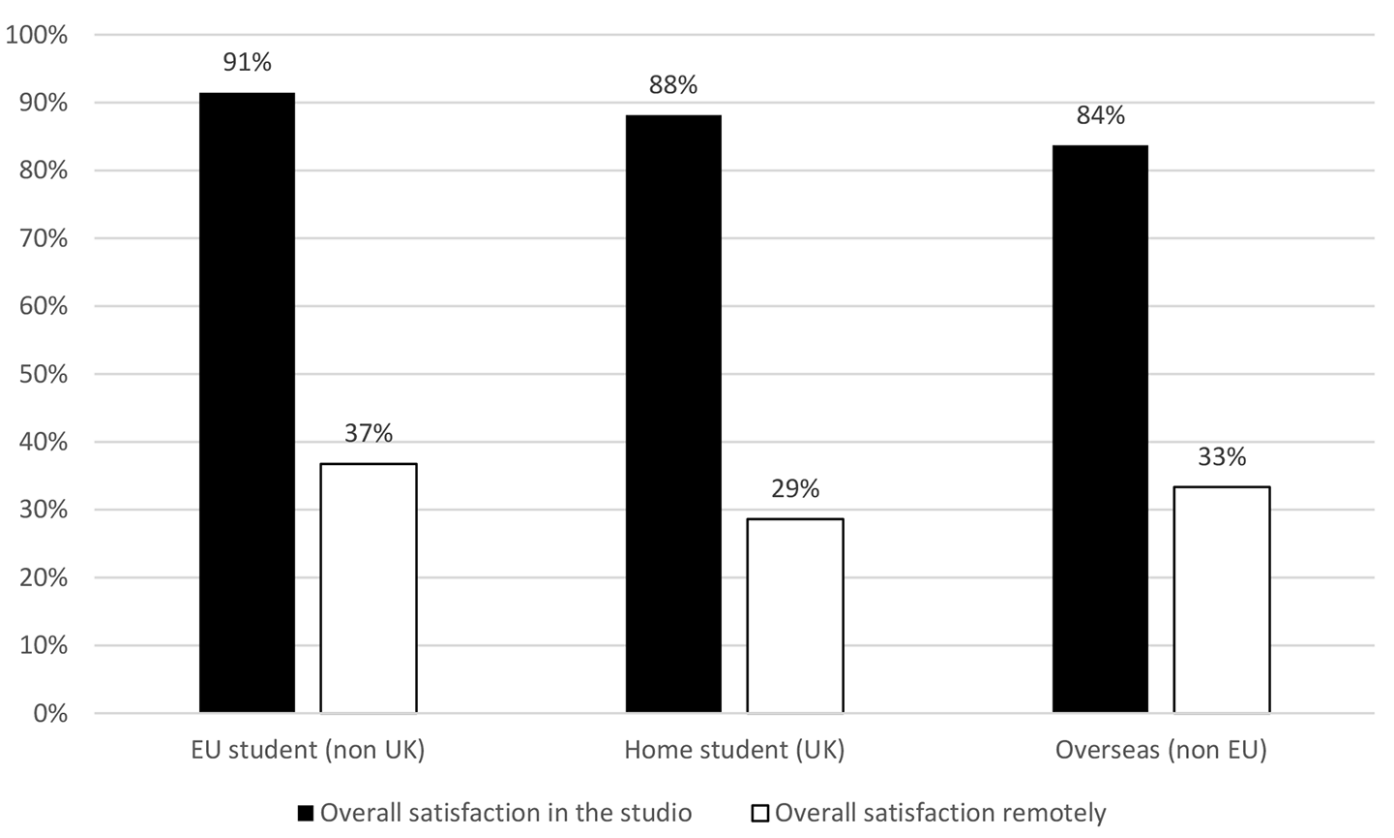
Change in overall satisfaction in different student statuses

**Fig. 8:**
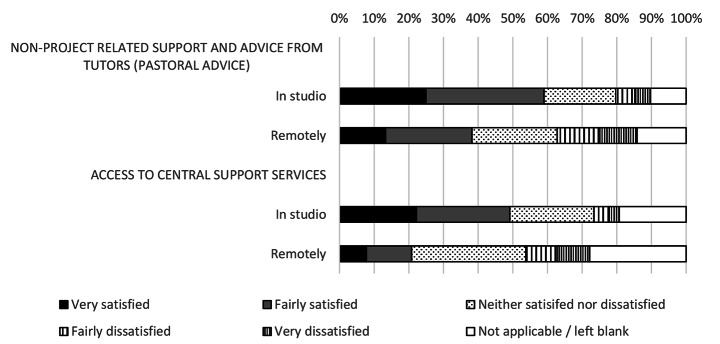
Student satisfaction with pastoral support mechanisms

### Impacts on mental health and well-being

Pastoral support was also widely impacted (Fig. 8). Students showed a 36% fall in relative satisfaction with pastoral support from tutors and a 57% drop in satisfaction with central support services, including wellbeing support. Tutors all showed a 55% fall in the perceived ease of assessing student wellbeing and a 53% relative fall in ease of providing pastoral support.

18% of students reported impacts on the personal health and well-being as a significant disadvantage from remote teaching. 11% described how the move to working from home had significantly reduced their motivation, productivity or focus. 2% of students directly referred to the impact on their mental health in free text comments, while 4% cited isolation as a major disadvantage. Other impacts included lack of exercise, the challenges of transitioning environments, impacts on routine and time management and a loss of confidence.

Spending time at home or with family was the biggest advantage for 6% of students. Lifestyle improvements (such as more exercise or a better diet) was cited by 4% of respondents. 5% of students found working remotely meant they were more relaxed or had more rest. 6% of students reported lower levels of stress, anxiety or pressure as being the biggest advantage.

19% of tutors reported practical and personal disadvantages of moving to remote studio working. These included the work being more time consuming, more stressful, poorer working environment and too much screen time. Other issues included a lack of flexibility, exhaustion, privacy, home distractions, staff isolation, mental health and cost.

**Table 6:** Representative quotes on health and wellbeing

Student comments	Staff comments
“[The biggest disadvantage is] my mental health deteriorating resulting in less motivation to work and not being able to do anything about it because it got brushed off as “we’re all in this together so let’s just give everyone the same extension cuz [sic] we’re all the same” (1st year, Part 2 student) “My mental health has massively deteriorated, my understanding of what I’m doing has dropped and I am in a constant state of confusion.” (2nd year, Part 1 student) “At uni I had people around me to help with things I find difficult because I’m registered blind.” (1st year, Part 1 student)	“The disintegration of the studio has been heart-breaking - it’s such a fundamental part of architectural education. I have first-hand witnessed the negative effects of students working in isolation.” (MArch tutor) “Student mental health is a big issue; I believe in architecture more than most fields of study. This is bound to be worsening because of remote working. When I ask students if they video chat with other students, the answer is generally no.” (Undergraduate and MArch tutor)

### The practicality and convenience of remote learning was considered its biggest advantage

Both students and staff reported some practical aspects of remote teaching and learning as being advantageous. These included no need to commute, savings in cost and time, flexible working and enhance work life balance. 32% of students mentioned how the move to remote studio had advantages in terms of practicality and convenience. Not having to commute meant many students could save time and money. For some it liberated time in their day allowing them more time for relaxation, hobbies, or spending time with families. Other students were able to save money through being at home, either by living with family to reduce living costs or avoiding spending when at the university. 12% of students found the physical environment they were working in to be preferable to the studio. For many it was more comfortable or convenient and avoided having to move equipment or work to and from the studio.

24% of students identified improvements in their lifestyle as a key opportunity of remote working. This included greater time efficiency, cost savings, reduced travelling and commuting and a better work-life balance. Several students relished the chance to be at home or with their family. Others suggested lower stress, improvements in physical health and living more sustainably were all opportunities for remote working.

**Fig. 9:**
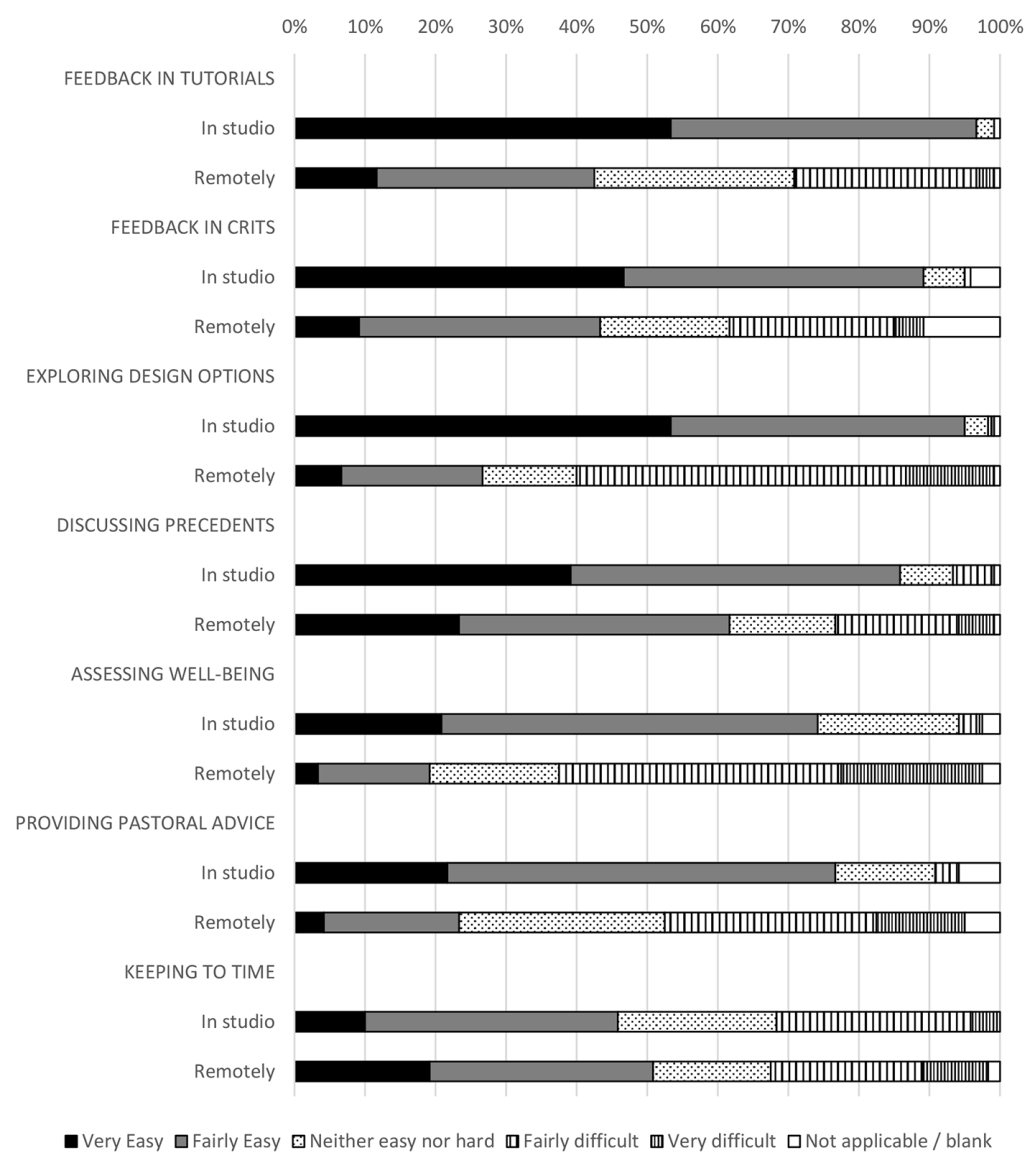
Perceived ease of educational delivery by tutors in the architecture studio and working remotely

The most cited advantages for staff were themed around the practical improvements that remote working offered. This was mentioned by 54% of staff. 28% of staff described not having to commute as one of the biggest advantages while others mentioned time management (6%) and flexibility to organise and undertake their teaching (8%). Other factors described by five or fewer tutors included the lower carbon footprint, more efficient teaching, convenience, structure, their home or office working environments (both physical comfort and with fewer distractions), the ability to learn digital skills, the students’ pre-submission of work (easing organisation) and the expansion of geographic limits that remote working offered. Overall, however, staff found educational delivery more challenging remotely (Fig. 9).

**Table 7:** Representative quotes on the practical advantages of remote working

Student comments	Staff comments
“Time taken to commute can be used to work more on projects.” (2nd year, Part 1 student) “Dedicated workspace and more time to focus on my work as there has been no commute (I previously had a 1 hour drive to my university due to my workplace location and being on a collaborative course). Having that time back has been great for my study and home life balance.” (2nd Year, Part 2 student) “Being in control of your work hours / schedule. Nice, quiet and comfortable environment (reduced social anxiety) Not having to spend money on expensive food and beverages in university.” (2nd year, Part 2 student) “The comfort and relaxed environment of working from home and reduced pressure often felt in studios.” (2nd year, Part 2 student) “[Working from home is] a bit more relaxed. It has helped with a healthier diet lifestyle unlike working in campus.” (2nd year, Part 1 Student)	“[The biggest disadvantage was] simply talking to the students and gauging their progress or asking how they are. Being able to motivate my students verbally, as sometimes emails can be taken in different ways. I found video calling uncomfortable.” (Undergraduate tutor) “Exhaustion. 8 + hr days of focussed 1 to 1 teaching are physically tiring in a way in studio teaching isn’t - this is draining and affects working and life on non-teaching days.” (Undergraduate and MArch tutor) “Stilted/more protracted communication methods with students in tutorials; everything takes longer. Not being able to get ‘the big picture’ by being able to ‘see’ a whole project on the wall in one go. The shift in presentation mode part way through the year from ‘on the wall exhibition’ to a digital submission has been hard for staff and students to transition to.” (Undergraduate tutor) “It actually became easier to carry out tutorials as groups were given a slot to adhere to, rather than an ad hoc approach in the studio in which often many students wanted a long time to chat rather than keep it concise and to the point.” (Undergraduate tutor)

### The quality of student and staff interactions was compromised

**Fig. 10:**
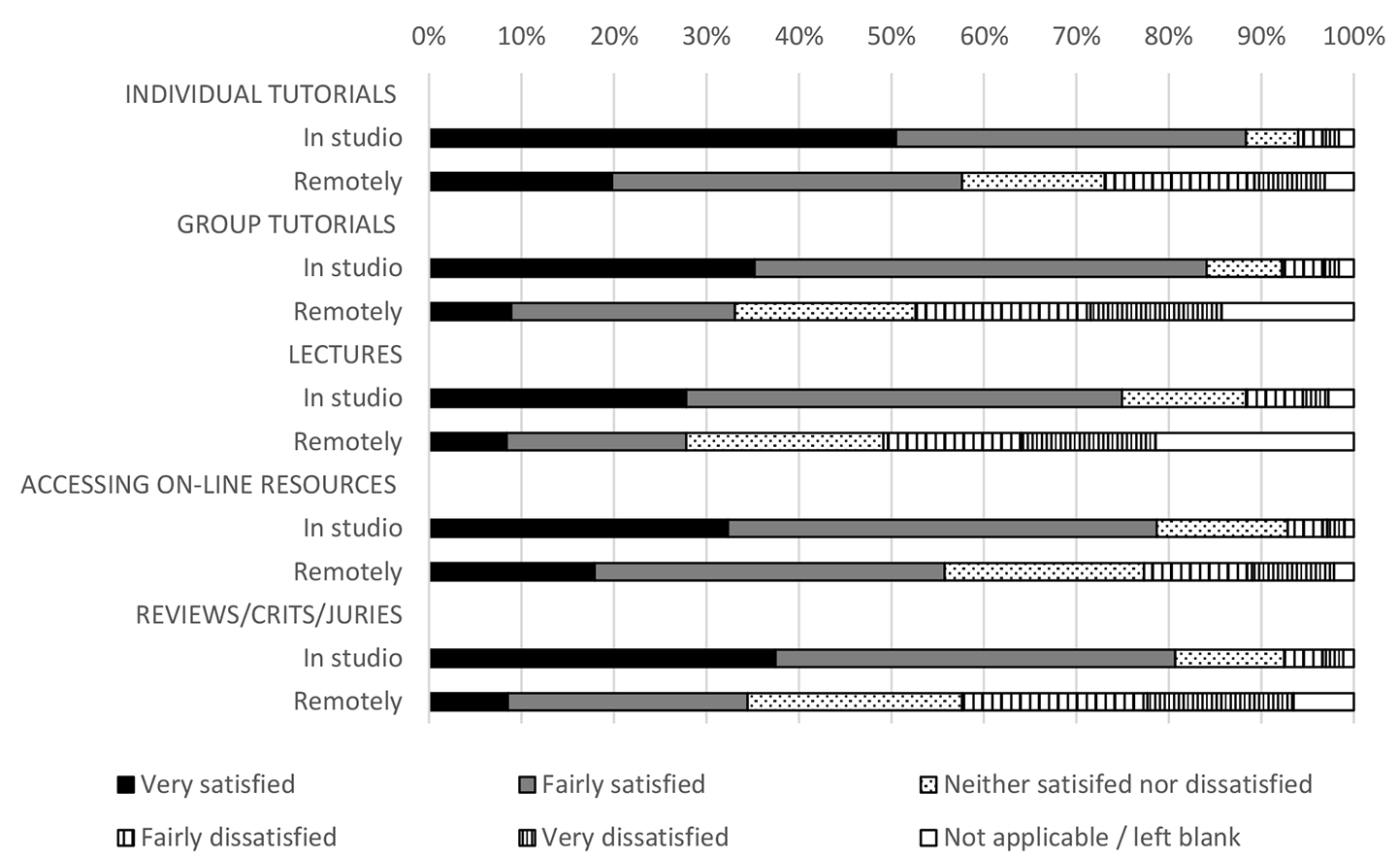
Student satisfaction with learning opportunities in the architecture studio and working remotely

**Fig. 11:**
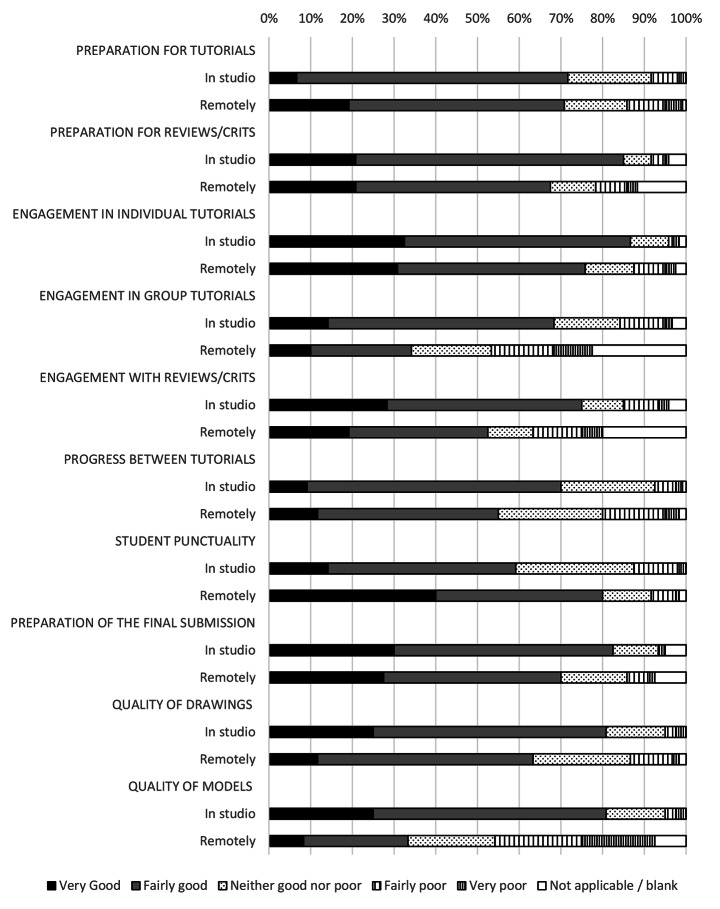
Tutors’ perceived quality with student engagement in the architecture studio and working remotely

Both students and staff perceived the quality of formal teaching interactions (reviews, juries, crits and tutorials) to have fallen after the move to online teaching (Figs. 10 and 11). Staff highlighted a concern with the nature of these interactions. Being able to “teach through drawing” was considered essential to architectural tutorials and significantly compromised through the move to remote teaching. Establishing relationships with students was also seen to be adversely affected by the move, which was particularly reflected in the responses from staff.

43% of staff described how their interactions with students had been compromised since the move to remote learning. 22% wrote about a sense of disconnection and lack of face-to-face encounters was a major disadvantage. 10% directly described how communication with students was more challenging remotely. Other comments included a perceived lack of student engagement, the inability to assess student’s capabilities and a focus on linear or didactic teaching methods. Staff also described how group tutorials, pastoral care, student parity and student support had all been compromised.

**Table 8:** Representative quotes about the quality of staff and student interaction

Student comments	Staff comments
“I think the studio days are really not working well and many tutors are not technically advanced enough.” (3rd year, Part 1 student) “[The biggest challenge is] not [being] able to have proper crits or presentations in person, can’t present physical models effectively.” (2nd year, Part 1 student) “[The biggest challenge is to enable] contacting/speaking to tutors in depth at any point, not having to schedule a meeting.” (3rd year, part 1 student)	“Not all students attended tutorials, perhaps a more flexible approach would work here.” (Undergraduate tutor) “[The biggest disadvantage is the] the lack of stretched discussions and sharing opinions in small groups discussions.” (MArch tutor) [The biggest disadvantage is] the resultant static interaction with each student. i.e. not being able to draw and discuss fluidly, but instead having to assess student work for each individual tutorial in advance and then wrestle with the confines of technology.” (Undergraduate tutor) “Students are more prepared for weekly tutorials [after the move to remote teaching] and give better presentations.” (Undergraduate tutor) “[The advantages are] too many to count. I am only a few years off retirement and am not a digital native…but I have found on-line tutorials very positive and easier than face to face (though I miss seeing students and colleagues in social terms). Students come better prepared, I can draw directly on work non-destructively, I can upload images of precedents directly into real-time files, students engage better in group tutorials – it’s all good in my experience.” (Undergraduate and MArch tutor)

### Enabling access to physical and digital resources for students and staff is essential for the success of remote teaching

Staff and students reported that providing access to digital and physical resources would significantly enhance their remote learning experience. This included access to hardware and the necessary software that previously would have been provided on campus. Staff frequently articulated the need for the resources and training to replicate in person teaching methods, particularly being able to draw remotely.

39% of students cited practical issues with working remotely. Lack of access to physical or digital resources was a major concern (cited by 29% of students). 12% reported that their working environment was unsuitable or impractical. Smaller numbers mentioned the absence of non-digital working was a big disadvantage and 6 students of 798 said technical issues (internet connection, software problems etc.) was a significant issue. 23% of respondents identified these concerns as a major challenge for remote learning in the design studio. Other challenges included workspace, “over management”, staff mental health, fatigue, personal commitments, staff training, technical issues and extended time for preparation and interactions.

31% of students said that accessing resources and facilities would be a challenge. This included access to digital resources and physical facilities (such as workshops), accessing suitable spaces to work and technical knowledge as a challenge to operate in a remote learning environment.

For staff, 23% cited personal or practical challenges. Inadequate equipment and resources were most frequently mentioned.

**Table 9:** Representative quotes about resource access

Student comments	Tutor comments
“…it is unfair to assume we all have the resources to do this. The difference in access to resources creates inequality in terms of the work we are able to produce.” (1st year, Part 2 student) “[The biggest disadvantage is] no resources or space to work. No laptop or computer so very difficult to do work I would normally do using the university computers.” (1st year, Part 1 Student) “…if we do not have the software at home it is a massive handicap, from talking to a few people this has massively effected the output of their final work.” (1st year, Part 2 student)	“[The biggest challenge was] teaching and learning by doing, particularly drawing and design process skills.” (Undergraduate and MArch tutor) “The frustration of not being able to draw and the length of time to properly communicate design feedback virtually.” (MArch tutor) “The intensity of the teaching seems to have increased. The immediacy of response - this is slower, partly because I do not have all of the kit that I need for responsive studio teaching i.e. Wacom tablet and pen, additional larger monitor, and printer/scanner. Typing feedback takes so much longer along with incorporating drawings - very time-consuming.” (Undergraduate and MArch tutor)

## Discussion

The results demonstrate a near ubiquitous view of staff and students of archtiecture in the UK of the essential nature of the design studio. Yet, while the removal of studios had an overwhelmingly negative impact on practical delivery of programmes, it was the cultural, pastoral and communal aspects that were most keenly felt by staff and students. The inability for students to learn from each other, seeing the work of others and sharing skills and knowledge, was directly challenged in a remote environment. This was echoed by tutors who perceived large impacts on the quality of all student interactions measured, such as working in teams and receiving technical help. “Studio culture” is a frequently cited term (Ward, 1990; Koch, 2002; Vowles et al., 2012) often associated with intangible social networks and “hidden curricula” that are built throughout architectural education (Dutton, 1987; Ward, 1990). It was mentioned widely in the survey from both staff and students. The physical design studio enables immediate moral support, benchmarking, and peer learning that may be contributory factors to developing an effective studio culture.

The quality of teaching interactions also suffered in the move to online learning however, there was an asymmetry observed between students and staff. Tutors perceived much larger negative impacts on the quality of these interactions. While their assessment of verbal feedback from individual tutorials was comparative to the student response factors such as the relative ease of exploring design, options was considered far more challenging. Even tasks which may have been enhanced by closer interaction with a computer (such as exploring design precedents) were also considered more challenging remotely by tutors. The one-to-one tutorial, often held at a desk or over a drawing board commonly operationalises drawing as a process between tutor and student in which the tutor acts intuitively often through drawing (Schön, 1985). Despite robust pedagogic critiques of this mode of teaching (Webster, 2004) teaching through demonstration and imitation is a primary tutor characteristic (Webster, 2004). For the tutor, demonstrating through pointing, manipulating a model, sketching or tracing are ways in which they might articulate their feedback (Goldschmidt et al., 2010). Despite technical “fixes” (tablets and stylus combinations or visualisers) the limitations of digital communications made the beneficial use of these nuanced interactions between staff and students far more challenging to achieve. Studies have suggested a student preference for a more reflective approach (Quinlan et al., 2007), which might explain the misalignment between student and staff responses, the tutors placing greater emphasis on the transmissive qualities of these interactions.

For many students, the move to remote working did not significantly alter satisfaction with their working environment. Yet those aspects of the studio space which arguably characterise its subject specific nature (the amount of layout space and access to technical resources particularly) suffered a far greater impact. Descriptions of the design studio frequently note the space given to students and the ability to make a mess, to personalise their working environment and scope to create objects at scale (inter alia Shaffer 2003; van Dijkum and CFDM, 2013; Corazzo 2019). Teaching staff considered the move to online learning particularly beneficial to acoustics and noise control in their workplace, which suggests deficiencies in these aspects of the studios where they typically worked.

The survey highlighted challenges in supporting student wellbeing and offering pastoral care. Mental health and well-being are of particular concern in architectural education with rates tripling in the last five years (Mirza & Nacey Research for the RIBA, 2020). While in part this may be due to structural and cultural challenges (for example the implicit expectations of staff for students to overwork) a supportive environment in which staff are facilitated to support students and recognise potential issues can be fostered. The move to remote learning made connections harder to forge and support harder to administer. The familial and home-like qualities instilled by the physical design studio (McClean, 2009) were broken, severing students’ social connections and the support and motivation this offers.

There were no significant differences in the different demographic groups surveyed in overall satisfaction, either by gender, ethnic group or student status. Within the free text comments however, both students and staff described how access to technical resources was a major limiting factor of working remotely. The survey did not capture economic status, and without further research no conclusions can be drawn on the impact of “digital poverty”. However, findings by (Summers et al., 2022), corroborates suggestions that students from more disadvantaged backgrounds had a larger drop off in engagement. While the picture may be more nuanced than only “digital poverty” evidence in the free comments suggests for some students, space to work and access to a computer or specialist software was a key barrier. This would have resulted in significant inequalities across the student body with respect to access to resources compared to the broad equality offered by a universal studio provision. It is likely the learning opportunities and academic output for some students were disproportionally diminished because of the removal of physical studios.

## Conclusions

It is hard to exaggerate the clear dissatisfaction with the emergency remote learning necessitated by the Covid-19 pandemic for architecture students. It may also suggest that educators were unable to capitalise on some of the potential opportunities of genuine online learning due to the speed at which the changes were introduced. The characterisation of architecture’s signature pedagogy by both its tutors and students as implicitly place based, spatial and in-person, has arguably framed the responses to the survey. The tradition of the “studio culture” is so deeply embedded in the identity of the profession that it may lead to both students and tutors being resistant to the sort of radical change that was implemented at speed and through necessity because of a national “lock down”.

The survey findings illustrate the value of building rich learning communities when providing online education; enabling peer-to-peer learning and support should be at the forefront of any fully digital approach to design education. It also suggests areas of relative success upon which educators might take advantage. For example, equipping and training staff to operate effectively in tutorials remotely, is an area in which some minor changes might offer significant improvements.

Attempting to translate a studio-based pedagogy to an online equivalent is clearly problematic without careful design and planning. While some practical changes might improve the digital studio offer, to effectively deliver a remote architectural education, alternative pedagogies must be considered which embrace the opportunities that remote learning offers and deliver remedies to its associated problems. Not only does this include embracing new methods and techniques of teaching, but also reconceptualising architectural education to move away from a place-based pedagogy. It remains to be seen whether any alternative pedagogy can provide equivalent benefits to those seen in the successful situated design studio.

Perhaps the greatest challenge is to replicate the pastoral and social support implicit in the physical design studio. While face-to-face formal interactions might be loosely substituted in remote learning, the intangible, unstructured and spontaneous activity cannot so easily be replaced. Aspects of peer-to-peer support, which can happen with relatively few barriers or hinderances in a physical studio, were perceived as significantly lost in the move to remote teaching. The psychological benefits of physical proximity, human contact and interaction appear important elements in fostering a sense of studio community in which lasting relationships are forged. These relationships appear to play a significant part in students’ evaluations of the quality of their overall student experience. What endures from the university experience is often the relationships formed through intense studio-based activity, and that activity can also establish a pattern of creative working which persists, with individuals seeking to replicate it when creating their own design practice environments in later life. The place-based pedagogy, when supported by an array of associated facilities (such as workshops, wi-fi, security, working space etc.) creates a relatively level playing field of educational opportunity. When this is removed students with financial means are able to replace key aspects of it, whereas students without are disadvantaged. Students who rely more on various forms of academic peer or tutor support, or who are isolated from non-academic support networks, find the loss of studio brings with it the loss of social support, which adversely impacts their well-being and performance. Addressing this challenge in the virtual realm, without leaving students isolated, or without support, should be a priority for educators.

This research has impacts for all schools of architecture who educate in the tradition of the design studio. By understanding factors which are highly valued by students, as well as those most under threat from the potential closure of physical studios, educators might mitigate the impacts of both the pandemic and economically motivated studio closures.

Further work is planned to repeat this survey over several years. This will help disentangle reactive responses to the current pandemic and identify lasting trends and attitudes which may form a basis for more meaningful and lasting change.
